# Selection of Mechanical Fragmentation Methods Based on Enzyme-Free Preparation of Decellularized Adipose-Derived Matrix

**DOI:** 10.3390/bioengineering10070758

**Published:** 2023-06-25

**Authors:** Jiayi Feng, Su Fu, Jie Luan

**Affiliations:** Breast Plastic and Reconstructive Surgery Center, Plastic Surgery Hospital, Chinese Academy of Medical Sciences, Peking Union Medical College, Beijing 100144, China

**Keywords:** decellularized adipose-derived matrix, cell fragmentation, mechanical method, ultrasonication, homogenization

## Abstract

Background: The decellularized adipose-derived matrix (DAM) has emerged as a promising biomaterial for inducing adipose tissue regeneration. Various methods have been employed to produce DAM, among which the enzyme-free method is a relatively recent preparation technique. The mechanical fragmentation step plays a crucial role in determining the efficacy of the enzyme-free preparation. Methods: The adipose tissue underwent fragmentation through the application of ultrasonication, homogenization, and freeze ball milling. This study compared the central temperature of the mixture immediately following crushing, the quantity of oil obtained after centrifugation, and the thickness of the middle layer. Fluorescence staining was utilized to compare the residual cell activity of the broken fat in the middle layer, while electron microscopy was employed to assess the integrity and properties of the adipocytes among the three methods. The primary products obtained through the three methods were subsequently subjected to processing using the enzyme-free method DAM. The assessment of degreasing and denucleation of DAM was conducted through HE staining, oil red staining, and determination of DNA residues. Subsequently, the ultrasonication-DAM (U-DAM) and homogenation-DAM (H-DAM) were implanted bilaterally on the back of immunocompromised mice, and a comparative analysis of their adipogenic and angiogenic effects in vivo was performed. Results: Oil discharge following ultrasonication and homogenization was significantly higher compared to that observed after freeze ball milling (*p* < 0.001), despite the latter exhibiting the lowest center temperature (*p* < 0.001). The middle layer was found to be thinnest after ultrasonication (*p* < 0.001), and most of the remaining cells were observed to be dead following fragmentation. Except for DAM obtained through freeze ball milling, DAM obtained through ultrasonication and homogenization could be completely denucleated and degreased. In the in vivo experiment, the first adipocytes were observed in U-DAM as early as 1 week after implantation, but not in H-DAM. After 8 weeks, a significant number of adipocytes were regenerated in both groups, but the U-DAM group demonstrated a more efficient adipose regeneration than in H-DAM (*p* = 0.0057). Conclusions: Ultrasonication and homogenization are effective mechanical fragmentation methods for breaking down adipocytes at the initial stage, enabling the production of DAM through an enzyme-free method that facilitates successful regeneration of adipose tissues in vivo. Furthermore, the enzyme-free method, which is based on the ultrasonication pre-fragmentation approach, exhibits superior performance in terms of denucleation, degreasing, and the removal of non-adipocyte matrix components, thereby resulting in the highest in vivo adipogenic induction efficiency.

## 1. Introduction

Decellularized adipose-derived matrix (DAM) has become a research focus in the field of tissue engineering. This is because it has been shown to control cell behavior and accelerate tissue regeneration. Its ability to induce adipogenesis in vivo and in vitro has been widely confirmed [1,2,3]. As a potential tissue-specific regeneration scaffold, it has a broad application perspective in reconstruction and plastic surgery [3,4,5]. The classical adipose tissue decellularization process mainly involves mechanical crushing, enzymatic digestion, elution of the cationic agent, degreasing, and so on [1,2,3,4,5]. However, the use of biological enzymes for decellularization is time-consuming and expensive on the one hand. On the other hand, the enzyme protein (collagenase or trypsin) may activate complement and cause a local inflammatory reaction, so it is banned for clinical use in products in many countries [6,7,8]. More importantly, biological enzymes first digest and destroy the thin part of DAM, which greatly affects the yield and quality of DAM. In a previous study, Liu et al. [9] and our team [10] found that by increasing the intensity of mechanical disruption, the cellular components in the adipose tissue can be effectively removed without using biological enzymes to obtain DAM. We called this preparation method the enzyme-free method. Its main principle is to directly fragment most of the cells through a variety of mechanical fragmentation methods, so that the nucleus and other immunogenic components can be easily eluted, which completely cancels the effect of enzymes. Therefore, the efficiency of mechanical fragmentation is the key to determining the quality of decellularization without enzymes.

Currently, the most commonly used mechanical methods for tissue fragmentation are ultrasonication, homogenization, and freeze ball milling. For freeze ball milling, the grinding ball is used as a medium, to grind the tissue and cells by impact, extrusion, friction, and other methods. The low temperature ensures that the protein activity associated with the extracellular matrix is protected. In addition, the working principle of the homogenizer is to crush all cells and fibers with high shear force, while the ultrasonication acts almost only on the cells. The adipocytes could be crushed exclusively, while the fiber components were hardly affected [11,12,13]. Nevertheless, there are few studies on the fragmentation efficiency and characteristics of adipocytes by different mechanical methods. In this study, we aim to compare the effect of fragmentation of adipocytes by three mechanical comminution methods to optimize the quality of DAM and the efficiency of adipogenic induction in vivo.

## 2. Methods

### 2.1. Fragmentation of Adipocytes

Fresh human adipose tissue was harvested from 5 healthy female patients aged 28–43 years who underwent negative pressure liposuction of the abdomen or thighs at the Plastic Surgery Hospital of the Chinese Academy of Medical Sciences. All protocols using human samples were approved by the Ethics Committee of the Plastic Surgery Hospital (NO. ZX201843), and samples were collected with written informed consent.

The remaining adipose tissue was collected and transported to the laboratory within 2 h at low temperature. Fresh adipose tissue was stratified after standing for 15 min. After removing as much blood and swelling fluid as possible, the coarse fibers of the adipose tissue were picked out with sharp forceps. The remaining fat was washed three times with sterilized distilled water. The following 3 crushing methods were used to break 20 mL cleaned fat (*n* = 3) each time, and the parameters were all referenced to the optimal parameters specified by the device manufacturer. (1) Ultrasonication: ultrasonication instrument (JY96-IIN, Huxi Ltd., Shanghai, China) was used 3 times with a power of 90 W under stirring in an ice bath. The operation time was 3 min each time. The ultrasound was turned on for 1 s and off for 2 s. (2) Homogenization: a high-speed dispersion homogenizer (FJ200-SH, Huxi Ltd., Shanghai, China) was used for comminution at 20,000 r/min × 2 min. (3) Freeze ball milling: Freeze ball grinding machine (JXFSTPRP-CLN, Jingxin Ltd., Shanghai, China) was used 6 times for crushing at 60 Hz and −15 °C. Each grinding time lasted 1 min, and the interval was suspended for 20 s.

### 2.2. Immediate Comparison of Crushed Adipocytes

After crushing in three methods, the center temperature of the mixture was immediately measured and recorded using a high-precision thermometer (DT1311, Lihuada Ltd., Shenzhen, China). After removing possible coarse fibers, the suspension treated by the 3 methods was placed in a 50 mL centrifuge tube and centrifuged at 1000 rpm for 5 min. After centrifugation, the top layer (oil layer) was aspirated to measure the volume, and the thickness of the middle layer (residual fat layer) was also measured.

An appropriate amount of the interlayer tissue was poured onto sterile gauze to remove the water in the tissue. Fluorescent staining was performed using Bodipy, PI, and Hoechst to evaluate the activity of the remaining cells immediately and the cell membrane damage after the tissue was crushed. Hoechst 33342+ BODIPY+ PI (Thermo Fisher Scientific, Waltham, MA, USA) was prepared, shaken in the dark for 30 min, and purified twice with PBS. A 30 μm 3D image was reconstructed under a laser scanning microscope (Leica, Allendale, NJ, USA).

An appropriate amount of the interlayer tissue was removed for fixation, gradient drying, and desiccation. After 30 s of gold spraying, images were acquired with a scanning electron microscope (SU8100, HITACHI Ltd., Tokyo, Japan) to observe the microscopic features of broken adipocytes and adjacent extracellular matrix in different fragmentation methods.

### 2.3. Preparation of Decellularized Adipose-Derived Matrix in Enzyme-Free Method

The remaining sediments were modified to produce DAM based on our original enzyme-free decellularization method [10]. Simply put, the sediments were soaked in a 1% Triton X-100 solution in a shaker for 48 h and then rinsed 5 times with sterilized distilled water at 10 min, 30 min, 1 h, 2 h, and overnight intervals (>12 h). The remaining precipitate was shaken in isopropyl alcohol at constant temperature (37 °C, 100 rpm) for 6 h to remove the remaining lipids, and then rinsed 3 times with sterile distilled water, 30 min each time. The next step was to add 75% alcohol and rinse 3 times for 30 min each, then add sterilized distilled water and rinse 3 times for 30 min each. The wet DAM was stored in 1% penicillin–streptomycin solution at 4 °C.

### 2.4. Characteristics of DAMs

The DAM obtained by three mechanical fragmentation methods were generally observed. They were fixed with 10% buffered formalin, with some of the DAM embedded in paraffin, and then sliced and stained with HE to observe the histological features and to determine if nuclear remnants were present. Some were frozen and then stained with Oil Red O to see if nuclear and lipid remnants were present. After the DAMs were freeze-dried in a vacuum freeze-drying machine, the dry weight of the DAM was measured. The DNA extraction kit (Solarbio Science & Technology Co., Ltd., Beijing, China) was used to extract DNA according to the protocol. The residual DNA concentration was determined using NanoDrop 2000 (Thermo Fisher Science, Waltham, MA, USA) (*n* = 3).

### 2.5. In Vivo Transplantation Experiment

BALB/c-nude male mice aged 6–8 weeks were used in the experiment. The animal experiments were approved by the Ethics Committee of the Plastic Surgery Hospital [2023(2)]. The procedures for animal experiments strictly followed the regulations and standards for the protection and use of experimental animals formulated by the Chinese Academy of Medical Sciences and Peking Union Medical College.

DAM obtained from ultrasonication (U-DAM) and DAM prepared by homogenation method (H-DAM) were 50 μL for each injection site. They were then cut and thoroughly mixed with normal saline. An 18-gauge needle was used to inject the left and right sides of the back of the mice (*n* = 5 per group at each time point), respectively. The mice were euthanized between 1 and 8 weeks after injection to obtain implant samples.

### 2.6. Histological and Immunohistochemical Staining

After fixation in 4% paraformaldehyde for 24–48 h, samples were embedded in paraffin and sectioned. Hematoxylin and eosin (HE) were used to examine the morphology of the implants at each study time point. Immunohistochemical staining was performed for the lipogenic marker Perilipin-1 (Abcam ab3526, Cambridge, UK, 1:1000) and CD31 (Abcam ab28364, Cambridge, UK, 1:4000) was used to locate vascular endothelial cells. Sections were scanned using a microscopic digital slice scanning system (Motic EasyScan Pro 6). Image J software was used to calculate the area proportion of panoramic Perilipin-1-positive adipocytes to assess adipogenesis in implants. The CD31-positive area proportion of 5 random fields of view (×100 magnification) was calculated to assess angiogenesis in the implants in each group.

### 2.7. Statistical Analysis

All data were expressed as mean ± standard deviation (SD). Statistical analysis was performed using SPSS 26.0 (SPSS, Chicago, USA). Statistical significance of the data was analyzed at the 95% confidence level using analysis of variance. *p* < 0.05 is considered statistically significant (* *p* < 0.05, ** *p* < 0.01, *** *p* < 0.001).

## 3. Results

### 3.1. Effect of Adipocytes Fragmentation

**Figure 1:**
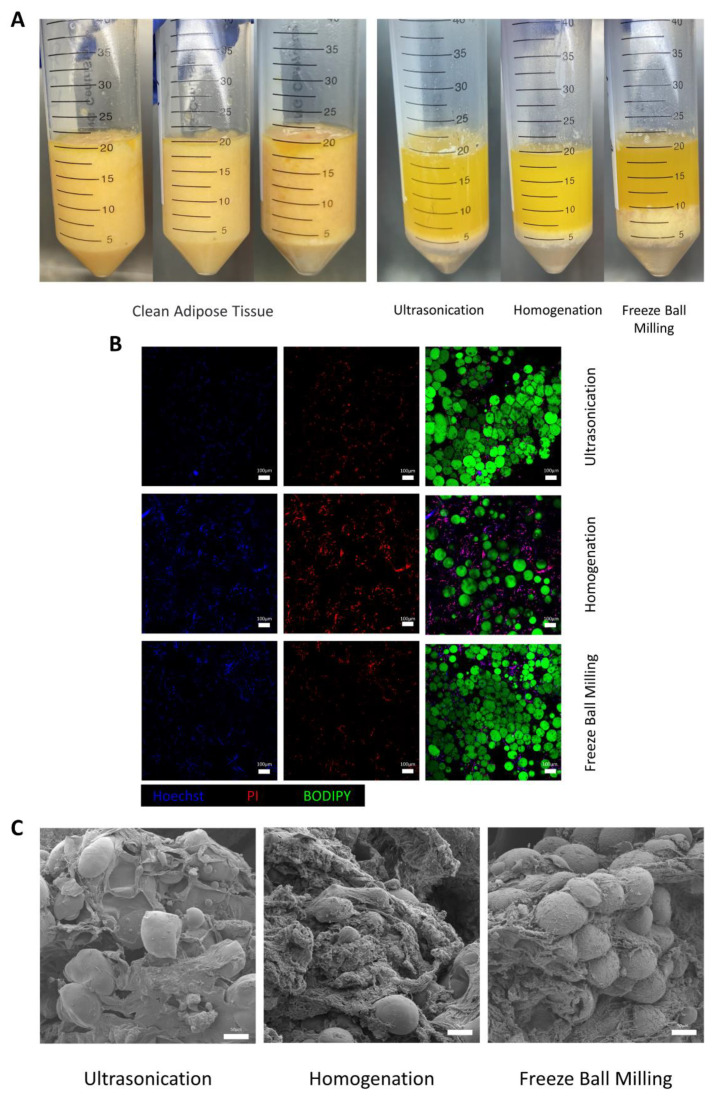
Characterizations of crushed adipose tissue. (**A**) (**left**) Adipose tissue before being broken. (**right**) Adipose tissue was Fragmentated by ultrasonication, homogenization, and freeze ball milling along with centrifugation. (**B**) Immediate fluorescence staining with Bodipy, PI, and Hoechst to assess residual cell activity. Green is for lipids, red is for dead nuclei, and blue is for living nuclei. Scale bar = 100 μm. (**C**) Scanning electron microscope (SEM) images of interlayer tissue after ultrasonication, homogenization, and freeze ball milling, showed broken adipocytes attached to matrix and fibers. Scale bar = 50 μm.

After the adipose tissue was fragmented by 3 methods, the suspension was divided into 3 layers after centrifugation (Figure 1A). The amount of oil in the upper layer, the thickness of the middle layer, and the immediate center temperature after fragmentation are detailed in Table 1. The oil discharge after ultrasonication and homogenization was not significantly different (*p* = 0.8813), but it was significantly higher than in the freeze ball milling group in both cases (*p* = 0.0007). The middle layer of ultrasonication was the thinnest (*p* < 0.001), which was significantly better than the other 2 methods. The immediate center temperature after fragmentation was lowest [(25.83 ± 0.55) °C] for the freeze-grinding method, (36.53 ± 0.70) °C in ultrasonication, and highest [(40.50 ± 0.70) °C] for the homogenization.

The interlayer tissue obtained from three fragmentation methods was subjected to fluorescent staining using Bodipy, PI, and Hoechst. PI selectively stains cells with damaged membranes. In Figure 1B, it is evident that Bodipy staining highlights the lipids in green, while a significant number of cell nuclei appear purple due to the overlapping staining of PI (red) and Hoechst (blue). Compared with ultrasonication and homogenization, the intercellular connections of the residual adipocytes were tighter in the freeze ball milling method. In addition, there was less residual nuclear material in the ultrasonication group, whereas a large number of dead red nuclei were densely seen in the homogenization group.

Scanning electron microscopy examination revealed a large number of fibers and some undestroyed adipocytes and cell fragments in the three crushing methods (Figure 1C). Ultrasonication and the freeze ball milling group still showed a tiny number of adipocytes wrapped in dense fibers, which, in contrast, hardly occurred in homogenization.

### 3.2. Characteristics in Three Types of DAMs

**Figure 2:**
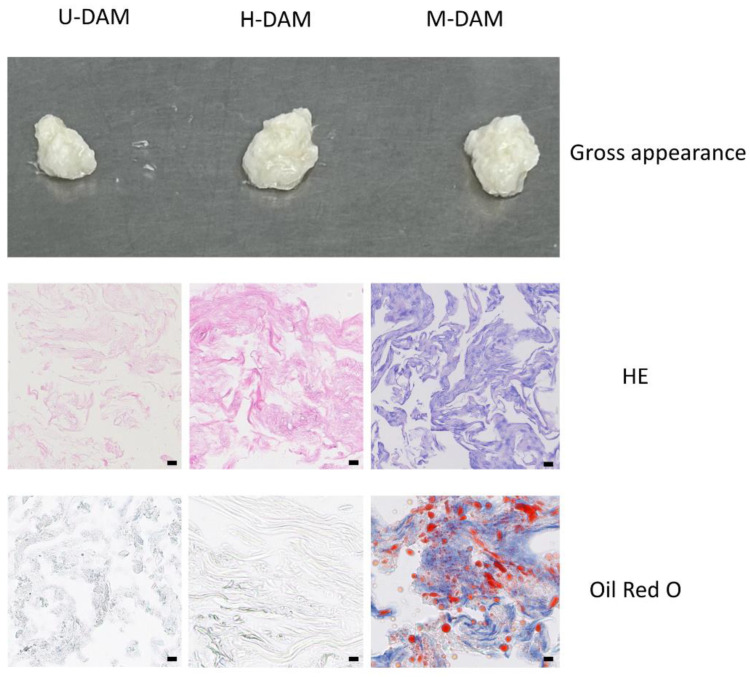
Characterizations of ultrasonication-DAM (U-DAM), homogenation-DAM (H-DAM), and freeze ball milling-DAM (M-DAM). (**top**) HE staining for (**middle**) three DAMs. Scale bar = 10 μm. (**bottom**) Oil Red staining for three DAMs. Scale bar = 10 μm.

The DAMs obtained by the three different crushing methods were all white flocculent solid (Figure 2A). In histological staining, the U-DAM showed a relatively uniform fine fiber structure, whereas the H-DAM and freeze ball milling (M-DAM) showed diverse fiber structures, most of which were fine fiber-like, while the inclusion fraction was coarse and swirly (Figure 2B). In both U-DAM and H-DAM, no red colored lipid components were found with oil red O staining (Figure 2C). However, in the M-DAM, the fibers were not only covered with blue-stained nuclei in the HE staining, but also the residues of red-stained lipids were observed in the oil red O staining. In the quantitative determination of DNA residues, the DNA residue of U-DAM was (28.93 ± 4.64) ng/mg and the DNA residue of H-DAM was (42.26 ± 8.01) ng/mg, both of which were in accordance with the standard for decellularized DNA residues in tissue [13]. In contrast, the DNA residue of M-DAM was as high as (7669.75 ± 1316.80) ng/mg, and the effect of denucleation and degreasing did not meet the standard. Therefore, only U-DAM and H-DAM were used for the subsequent in vivo experiments.

### 3.3. Adipogenesis by DAMs In Vivo

**Figure 3:**
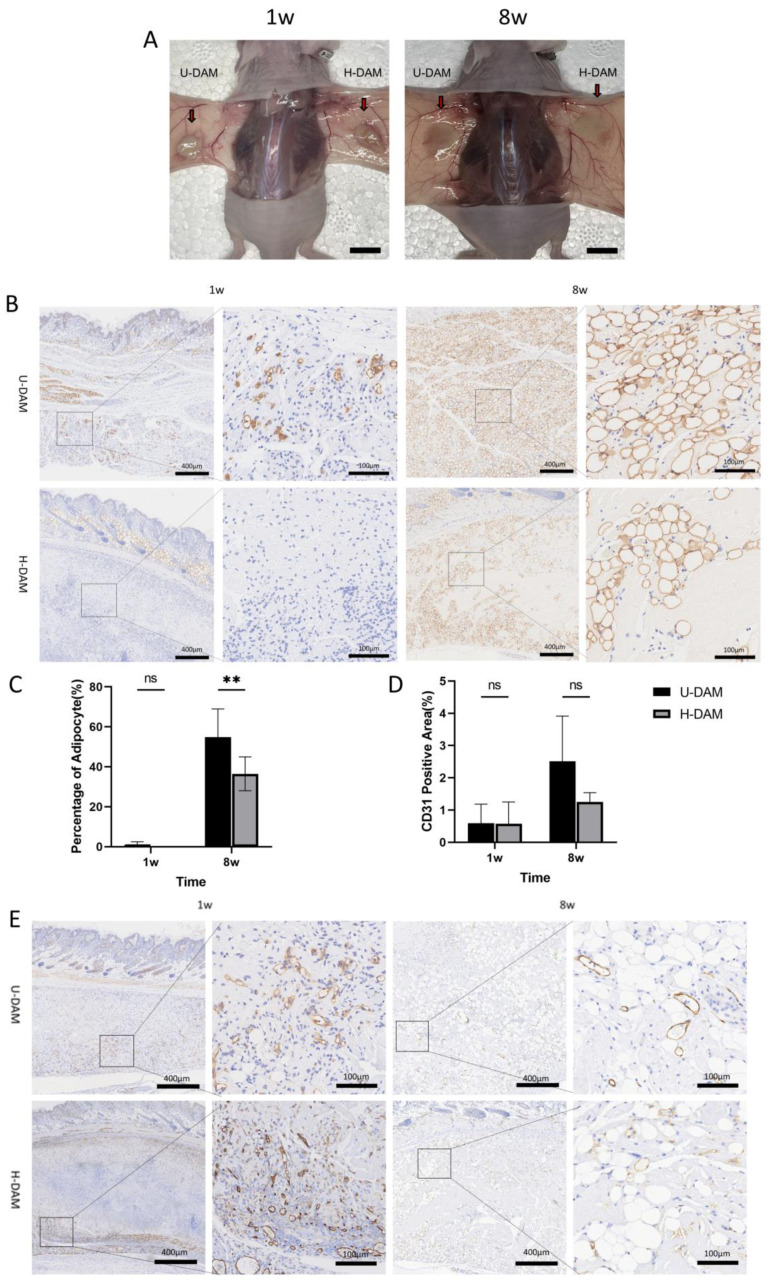
The regeneration of adipose tissue after the injection of U-DAM and H-DAM in BALBc-nude mice. (**A**) Implantation of 1 w U-DAM (**left**) and 8 w H-DAM (**right**) into the back of nude mice. Scale bar = 1 cm. (**B**) Perilipin-1 staining of adipocytes after U-DAM and H-DAM implantation. Scale bar = 400 μm, enlarged Scale bar = 100 μm. (**C**) Adipocyte area ratio after U-DAM and H-DAM implantation. (**D**) Vascular area ratio after U-DAM and H-DAM implantation. (**E**) CD31 staining after U-DAM and H-DAM implantation. Scale bar = 400 μm, enlarge Scale bar = 100 μm (ns: not significant. ** *p* < 0.01).

U-DAM was administered subcutaneously on the left side of the back, whereas the H-DAM was injected on the right side as planned. General observation showed that the implants on both sides had clear borders, certain thickness, similar shape and size, relatively white color, and soft texture during the first week (Figure 3A). At 8 weeks, the implants on both sides looked thinner and had a dark red color. Distinct small vessels were visible on the surface of the implants. Perilipin-1 staining (Figure 3B) showed clear infiltration of inflammatory cells at 1 week in both groups, which had largely resolved by 8 weeks. In the U-DAM, locally newborn immature multilocular adipocytes were observed in the first week (3 of 5), whereas no adipocytes were observed in group H-DAM. At 8 weeks, there was a large number of mature adipocytes in both groups. The regeneration of adipocytes in the U-DAM group was significantly more vigorous than in the H-DAM group at 8 weeks (Figure 3C) (*p* = 0.0057). In the U-DAM group, the matrix was almost filled with adipocytes, while in the H-DAM group there were still some empty matrix areas without adipocytes. When vascularization was assessed (Figure 3E), dense and small blood vessels were seen at the edge of the implants in both groups during the first week. Over time, the vessel diameter increased and became sparser at week 8, but there was no significant difference in DAM angiogenesis between the 2 groups at 1 week (*p* = 0.9991) or 8 weeks (Figure 3D) (*p* = 0.0627).

## 4. Discussion

The key to producing a decellularized adipose-derived matrix is first to break the cell membrane by various methods, then repeatedly elute the nuclear material with immunogenicity and a mass of lipid droplets inside the adipocyte. Thus, we could obtain a cell-free biological scaffold. Various cell-breaking methods are often used in the preparation of DAM to obtain the best results [14,15,16,17,18,19]. In general, decellularization methods can be divided into the enzymatic method (a variety of biological enzymes directly collapse the cell membrane and other corresponding target substances), the chemical osmosis method (using surfactants or stain removers to alter the permeability of the cell membrane so that the contents can selectively leak out), the mechanical method (mechanical disruption of the membrane), and the physical method (use of repeated freezing and thawing or osmotic pressure difference inside and outside the cell to break the membrane) [1,5]. The enzyme-free method is a combination of mechanical and chemical osmosis methods. Our results show that a small number of adipocytes in the middle layer cannot be completely broken after mechanical breaking alone, regardless of the mechanical method. To avoid the introduction of foreign enzymes and the possible destruction of extracellular matrix proteins, Triton X-100 solution was chosen as the method to further break the membrane. Our previous studies have shown that the DAM, prepared by the enzyme-free method, can be effectively denucleated and degreased [10].

There is no doubt that the mechanical method is the most critical step to determine the effect of the enzyme-free method to produce DAM. In this study, three common mechanical methods of tissue fragmentation, including ultrasonication, homogenization, and freeze ball milling, were used to compare the effect of adipocyte fragmentation and the difference in the effect of adipogenesis induced by DAM in vivo.

There are different types of grinding, such as extrusion, collision, friction, cutting, shearing, and so on. For samples with high hardness, collision, extrusion, friction, and other methods are generally used, while for soft ones, the cutting method is more efficient. The ball mill uses the grinding ball as the medium to crush the sample by impact, extrusion, and friction [20]. We also found that the ball mill is not ideal for grinding adipose tissue because a number of adipocytes are still unbroken from both the macro and micro levels. We believe that freeze ball milling is not suitable to be used as a pre-mechanical comminution method for DAM preparation without enzymes. At the same time, it should be noted that the stainless steel grinding ball carries the risk of crisps coming off during the grinding process, which will add other impurities to the DAM raw sample.

The homogenizer adopts the high-speed shearing and dispersion method to produce strong liquid shearing and high-frequency mechanical action, which can effectively shear, tear, and mix to achieve uniform dispersion [21]. The homogenizer has a good crushing effect on the high viscosity solid–liquid mixture with large particles, and is suitable for crushing adipose tissue [22,23]. Ultrasound converts electrical energy into sound energy and bursts the cells by the cavitation of sound waves [24,25]. However, at high frequency and high pressure, large amounts of heat can also be generated [26,27]. Without stirring or an ice bath to dissipate heat, the temperature of the fat suspension reached 70–80 °C during ultrasonication. Local overheating of the suspension can easily lead to thermal denaturation of the active protein and eventually affect the adipogenic effect of DAM. Therefore, the specially designed stirring ice bath device can well solve the problem of high temperature. With the same setting of ultrasonic parameters, the sample temperature can be maintained at (36.53 ± 0.70) °C.

Our study shows that ultrasonication is the best method for the pre-mechanical fragmentation approach based on enzyme-free preparation of DAM. This is because, on the one hand, ultrasonication had the best effect on adipocyte fragmentation in terms of oil production, interlayer thickness, and immediate center temperature, followed by homogenization. On the other hand, DNA residues and the effect of adipogenesis of U-DAM were also the best. Freeze ball milling can maintain the lowest temperature in the liquid center but has the lowest oil output and the least comminuted adipose tissue in the middle layer. Its crushing effect is not ideal, not to mention that the subsequent M-DAM cannot meet the denucleation and degreasing requirements. Previous studies have also shown that small blood vessels are abundant in such coarse fibers in lipoaspirates. Many of the adipose-derived stem cells were located near blood vessels in adipose tissue [28,29]. On the confocal image immediately after fragmentation, we can also see that there are many cell-free nuclear components on the remaining fibers after comminution by the homogenizer, but such a phenomenon was not found in the ultrasonication. We suggest that the residual nuclear material in the DAM is mainly on the blood vessel and fiber components rather than on the adipocytes themselves, resulting in the residual DNA amount of H-DAM being significantly higher than that of U-DAM. Both U-DAM and H-DAM can achieve a satisfactory lipogenesis effect in vivo. However, the fat regeneration rate of U-DAM was much faster than that of H-DAM, and the final adipogenesis induction effect was significantly better than that of H-DAM. We found that the adipocytes in U-DAM grew relatively evenly distributed in the 8 weeks, while in H-DAM there were some matrix areas where the adipocytes could not grow all the time and became the “adipocyte-free zone”. It may be the reason why the adipogenesis effect of H-DAM was inferior to that of U-DAM.

However, there are still some limitations on ultrasonication. The fragmentation time is longer than homogenization, and the process is more complicated.

## 5. Conclusions

As ultrasonication and homogenization are effective methods to completely break adipocytes at the initial step, DAM can be produced with an enzyme-free method that allows successful regeneration of adipose tissues in vivo. Moreover, the enzyme-free method based on the ultrasonication pre-fragmentation method has a better effect on denucleation and degreasing as well as the removal of non-adipocyte components of the matrix, resulting in the best in vivo adipogenic induction efficiency.

## Figures and Tables

**Table 1:** Effect of three mechanical fragmentation methods on adipocytes (*** *p* < 0.001).

	Ultrasonication	Homogenation	Freeze Ball Milling
Oil (ml)	13.93 ± 0.25	13.67 ± 1.10	10.10 ± 0.30 ***
Thickness of Middle Layer (cm)	0.20 ± 0.02 ***	0.39 ± 0.01 ***	1.20 ± 0.07 ***
Temperature (°C)	36.53 ± 0.70 ***	40.50 ± 0.70 ***	25.83 ± 0.55 ***

## Data Availability

The data that support the findings of this study are available from the corresponding author, Luan, upon reasonable request.
